# Association of renal function screening frequency with renal function decline in patients with type 2 diabetes: a real-world study in primary health care

**DOI:** 10.1186/s12882-022-02979-1

**Published:** 2022-11-04

**Authors:** Henry Sundqvist, Eveliina Heikkala, Jari Jokelainen, Giuseppina Russo, Ilona Mikkola, Maria Hagnäs

**Affiliations:** 1 Rovaniemi Health Center, Koskikatu 25, 96200 Rovaniemi, Finland; 2 Center for Life Course Health Research, University of Oulu, P.O Box 8000, FI-90014 Oulu, Finland; 3 Medical Research Center Oulu, University of Oulu and Oulu University Hospital, P.O Box 5000, 90014 Oulu, Finland; 4 Department of Clinical and Experimental Medicine, University of Messina, Messina, Italy

**Keywords:** Kidney Function, Primary Health Care, Screening Frequency, Type 2 Diabetes

## Abstract

**Aims:**

To examine the association of the screening frequency of estimated glomerular filtration rate (eGFR) with the substantial reduction in eGFR (≥ 25%) among type 2 diabetes (T2D) patients with normal (eGFR≥60 ml/min/1.73 m^2^) and impaired kidney function (eGFR< 60 ml/min/1.73 m^2^).

**Methods:**

A longitudinal study involving 5104 T2D patients with follow-up period of 6.8 years (1.9 SD) were treated at the Rovaniemi Health Center, Rovaniemi, Finland during 2011–2019. The association between the screening frequency of eGFR (yearly vs. non-yearly) and the substantial reduction in eGFR was studied with logistical models and adjusted with biochemical variables and preventive medications.

**Results:**

Among the T2D patients with normal kidney function, non-yearly eGFR screening was significantly associated with substantial eGFR reduction in both unadjusted (odds ratio [OR] 3.29, 95% confidence interval [CI] 2.54–4.33) and adjusted models (OR 2.06, 95% CI 1.21–3.73) compared with yearly screening frequency. In the group of patients with impaired kidney function in the unadjusted model, non-yearly eGFR screening was significantly associated with substantial eGFR reduction (OR 2.38, 95% CI 1.30–4.73), but became non-significant after adjustments (OR 1.89, 95% CI 0.61–7.21).

**Conclusions:**

This study underscores the role of regular eGFR screening in the prevention of kidney function decline.

## Introduction

Type 2 diabetes (T2D) is an alarming global pandemic that is affecting a steadily increasing number of people. In 2019, it was estimated that 463 million (9.3%) of the world’s adult population have diabetes, and the number is estimated to increase to 578 million (10.2%) by 2030 [1]. In high-income countries, diabetes is twice as common compared with low-income countries. For instance, in 2021, the age-adjusted prevalence of diabetes was estimated to be 10.7% in the United States [1].

It is well established that T2D is a major risk factor for a wide range of vascular diseases, including ischemic heart disease and stroke, as well as chronic kidney disease (CKD) [2, 3]. Furthermore, CKD alone largely accounts for the increased all-cause mortality in patients with T2D [4]. During the last three decades, disability-adjusted life years (DALYs) attributable to diabetes and CKD have increased, whereas DALYs attributable to myocardial infarctions and strokes have declined [5].

The prevalence of CKD in patients with T2D is estimated to vary between 27.9 and 58.6% [5–10]. Traditionally, CKD in patients with T2D is characterized by the manifestation of albuminuria, followed by a decline in kidney function marked by a reduction in the glomerular filtration rate (GFR), finally leading to end-stage kidney disease (ESKD) [11]. However, recent studies state that CKD, especially in patients with T2D, is mostly characterized by renal impairment without measurable albuminuria [11, 12]. Many underlying causes of this non-albumic renal impairment have been hypothesized, for instance, the increased use of estimated GFR (eGFR), increased prescription of renoprotective medication, and earlier identification of CKD [11, 12].

In patients with T2D, regular screening and monitoring of CKD are widely recommended [2, 13, 14]. However, the screening and monitoring of CKD among patients with T2D have been shown to be inadequate [12, 15]. Recommendations are geared toward the regular monitoring of CKD, although evidence of the optimal screening frequency is scarce [2, 13, 14]. The current guidelines concerning monitoring frequency are based on expert opinion and indirectly derived from observational and renoprotective medication research [2, 13, 14]. As such, more in-depth knowledge of the relevance of optimal screening frequency is required.

The effective prevention of complications, especially CKD, is highly important in patients with T2D [13]. Intensive blood pressure (BP) and glucose control are still regarded as the cornerstones of CKD prevention, together with prognostic and renoprotective medication, such as glucagon-like peptide-1 receptor agonists (GLP-1 RAs), sodium-glucose co-transporter-2 inhibitors (SGLT2i), and renin-angiotensin-aldosterone system (RAAS) blocking therapy [13, 16]. Despite adequate interventions, CKD is considered to be a progressive disease, and therapy aims to slow the decline of kidney function [17, 18]. Therefore, the early identification of CKD through regular screening and adequate treatment is important.

The associations between cardiovascular risk factor control (glycosylated hemoglobin A1 (HbA1c), systolic blood pressure (sBP)) and prevention of renal function decline are well established [2, 11, 13, 14, 16]. However, the screening frequency of eGFR as a possible individual predictive factor of renal function decline among T2D patients has not been previously investigated. Therefore, the aim of this longitudinal study was to examine the association of the screening frequency of eGFR with a substantial reduction (≥ 25%) in eGFR among T2D patients with normal and impaired kidney function.

## Methods

### Study population

This study was a part of the Rovaniemi Primary Care T2D Study, which is a registry-based real-world study in a primary health care setting. Rovaniemi is a city and municipality located in northern Finland with a total population of 62,000 people living in both urban and rural areas. The study population consisted of 5104 patients who had received a T2D diagnosis between November 1, 2011 and February 19, 2019 at the Rovaniemi Health Center, Rovaniemi, Finland. The T2D diagnosis was based on the International Classification of Disease (ICD- [10]) codes for T2D (E11.1–E11.9) or the equivalent T2D code (T90) from the International Classification of Primary Care (ICPC). Patients with at least two evaluations of eGFR with a minimum interval of six months were included in the study. The data were retrieved from patient records. In the analyses, the baseline was considered as the first measurement of eGFR and the follow-up as the last measurement of eGFR within each patient. The study period was defined as the time between the baseline and follow-up, varying individually.

### Data collection

#### Reduction of eGFR

The Chronic Kidney Disease Epidemiology Collaboration (CKD-EPI) equation formulation, which is based on age, sex, race, and serum creatinine level (μmol/l), was used to calculate the eGFR (ml/min/1.73 m^2^). A substantial reduction in eGFR was defined as a reduction of ≥25% between the baseline and the follow-up in accordance with the Kidney Disease: Improving Global Outcomes (KDIGO) guidelines in CKD evaluation and management [2]. The “no substantial reduction” category included all others except those with substantial eGFR reduction.

#### Screening frequency of eGFR

The mean value of eGFR screening frequency between 2011 and 2019 was calculated and then dichotomized as yearly (365 days or under) vs. non-yearly (over 365 days), in line with the current national screening frequency recommendations [13, 19]. The first category was used as a reference.

### Covariates

We assessed the sex, age, achievement of treatment goals of bioclinical variables (HbA1c, LDL, and sBP), and prescription of antihyperglycemic and cardiovascular medications (Anatomical Therapeutic Chemical, ATC codes): statins (C10AA), any long-acting insulin (A10AE, A10AC), GLP-1 RAs (A10BJ), SGLT2i (A10BK), and angiotensin-converting enzyme inhibitors (ACEi) (C09A) or angiotensin II receptor blockers (ARB) (C09C) as potential covariates [2, 11, 13, 14, 16]. The preferred source of BP data was the patients’ own home measurements. If home measurements were not available, the measurements performed by a healthcare professional during the consultation visit were used [20, 21].

Age was measured at the follow-up. Achievement of the treatment targets for HbA1c, LDL, and sBP was defined according to the national guidelines as follows: HbA1c < 53 mmol/mol, LDL < 2.5 mmol/l, and sBP < 135 mmHg [22]. This was estimated at the baseline and follow-up. The patients were divided into two groups in terms of all three variables: 1) had achieved the treatment target at both time points, and 2) had not achieved the treatment target at both time points.

Data on prescribed or renewed prescriptions of statins, any long-acting insulin, GLP-1 RAs, SGLT2i, and ACEi/ARB were collected from the national electronic prescription registry using ATC codes. Medication data (new prescriptions or prescription renewals) were further processed by calculating the midpoint of the study period for each patient. Baseline medication was defined as before whereas follow-up medication was defined as after the midpoint. If the prescription was valid at the midpoint, then it was assumed to be the same at the baseline and follow-up. Follow-up medication data were used in the logistic models.

### Descriptive variables

Body mass index (BMI) was calculated by dividing the patient weight in kilograms by the square of their height in meters (kg/m^2^) and considered a continuous variable. In addition to the previously mentioned bioclinical variables and medications, the following measurements were gathered from the patient records: hemoglobin (Hb; g/l), diastolic BP (mmHg), and medications (ATC codes), such as calcium blockers (C08CA), beta blockers (C07AB), diuretics (C03), metformin (A10BA02), gliptins (A10BH), glitazones (A10BG), sulphonylureas (A10BB), fibrates (C10AB), multiple daily insulin injections (A10AE or A10AC and A10AB), and ezetimibe (C10AX).

### Study protocol and data collection

The data were recorded as part of each patient’s routine control visits at the health care center or during other visits and collected and handled anonymously using patient IDs for scientific purposes. Being a registry-based study, no written consent from the patients was required, in accordance with current Finnish legislation. The Ethics Committee of Lapland Central Hospital, Rovaniemi, Finland approved the study protocol in May 2018.

### Statistical methods

Clinical outcome measures were presented as mean and standard deviation (SD) and categorical variables as proportions. Continuous variables were tested with the independent samples t-test, while the Pearson χ^2^ test was used to evaluate the difference between categorical values. A multiple-multivariable binomial logistic regression analysis with odds ratios (ORs) and 95% confidence intervals (CIs) was performed to study the potential associations between substantial eGFR reduction and the screening frequency of eGFR. The logistic models were stratified by the baseline kidney function as normal (eGFR≥60 ml/min/1.73 m^2^) and impaired (eGFR< 60 ml/min/1.73 m^2^) kidney function, as the screening frequency recommendations differ between patients with and without impaired kidney function [2]. All statistical analyses were performed using the R software version 4.1.1. R Core Team (2020). A *p*-value < 0.05 was considered statistically significant.

## Results

The T2D study population consisted of 5104 patients with a mean age of 70.0 years (SD 12.7). The characteristics of the study population are presented in Table 1. During the average of the 6.8-year follow-up period (SD 1.9), beneficial changes were noted in LDL, HbA1c, BMI, and BP. The average systolic BP value decreased by 12.4 mmHg (*p* < 0.001) in the total population. A significant reduction in eGFR (≥25%) was observed in 12.6% (*n* = 582) of the population. The total number of prescribed antihyperglycemic and cardiovascular medications increased during the study period, as well as the usage of GLP-1 RAs and SGLT2i.

**Table 1:** Baseline and follow-up characteristics of the type 2 diabetes study population. Data are presented as mean (SD), except for sex, eGFR reduction, and medication (given as percentages of the population)

	Baseline	Follow-up	*P*-value
n	5104	5104	
Male	2755 (54.0)	2755 (54.0)	
Female	2349 (46.0)	2349 (46.0)	
Age, years		70.0 (12.7)	
Study period, years		6.8 (1.9)	
Mean eGFR screening frequency, days	344.5 (195.7)	294.7 (118.8)	< 0.001
LDL-cholesterol, mmol/l	2.9 (1.1)	2.5 (1.0)	< 0.001
HbA1c, mmol/mol	51.0 (15.0)	49.1 (13.2)	< 0.001
eGFR, ml/min/1.72 m^2^	81.9 (19.2)	76.5 (22.2)	< 0.001
Plasma creatinine, μmol/l	79.5 (34.1)	85.5 (52.1)	< 0.001
Hemoglobin, g/l	141.4 (14.5)	137.3 (18.5)	< 0.001
BMI, kg/m^2^	30.3 (6.0)	29.8 (5.8)	0.004
Systolic BP, mmHg	148.3 (23.0)	135.9 (18.4)	< 0.001
Diastolic BP, mmHg	83.0 (12.4)	77.9 (11.2)	< 0.001
eGFR reduction < 25%, n (%)		4046 (87.4)	
eGFR reduction ≥25%, n (%)		582 (12.6)	
Any hypertensive medication	3029 (59.3)	4093 (80.2)	< 0.001
ACEi/ARB	2045 (41.9)	2861 (58.6)	< 0.001
Ca blockers	1350 (27.7)	1974 (40.4)	< 0.001
Beta blockers	1867 (36.6)	2289 (44.8)	< 0.001
Diuretics	1186 (23.2)	1688 (33.1)	< 0.001
Any diabetes medication	3029 (59.3)	4093 (80.2)	< 0.001
Metformin	2417 (47.4)	3283 (64.3)	< 0.001
SGLT2i	24 (0.5)	704 (13.8)	< 0.001
GLP-1 RAs	11 (0.2)	154 (3.0)	< 0.001
Gliptin	917 (18.0)	1321 (25.9)	< 0.001
Glitazone	11 (0.2)	18 (0.4)	0.265
Sulphonylureas	169 (3.3)	131 (2.6)	0.030
Any long acting insulin	811 (15.9)	1178 (23.1)	< 0.001
Multiple daily injections insulin therapy	366 (7.2)	709 (13.9)	< 0.001
Statin	2741 (53.7)	3479 (68.2)	< 0.001
Ezetimibe	243 (4.8)	370 (7.2)	< 0.001
Fibrates	23 (0.5)	23 (0.5)	1.000

Note: The *p*-values present the differences between the groups tested with t-test or χ^2^-test. *ACEi*, Angiotensin-Converting Enzyme; *ARB*, Angiotensin Receptor Blockers; *BMI*, Body Mass Index; *DPP-4*, Dipeptidyl-Peptidase-4; *eGFR*, estimated Glomerular Filtration Rate; *GLP-1 RAs*, Glucagon-Like Peptide-1 Receptor Agonists; *HbA1c*, Glycosylated Hemoglobin; *HDL*, High-Density Lipoprotein; *LDL*, Low-Density Lipoprotein; *BP*, Blood Pressure; *SGLT2i*, Sodium-Glucose co-Transporter-2 Inhibitors

At baseline, 87.4% (*n* = 4336) of the population had normal kidney function (Table 2). The patients with impaired kidney function were older (81.3 [SD 8.9] vs. 68.9 [SD 12.1] years) and more frequently screened for eGFR (252.9 [SD 111.7] vs. 300.1 [SD 118.7] days) compared with the patients with normal kidney function (*p* < 0.001 for both). A substantial reduction in eGFR was observed among 25.0% (*n* = 146) of the patients with impaired kidney function and 10.8% (*n* = 436) among those with normal kidney function (*p* < 0.001).

**Table 2:** Baseline characteristics of the type 2 diabetes study population categorized as having normal or impaired kidney function. Data are presented as mean (SD), except for eGFR reduction, and medication (given as percentages of the population)

	Normal kidney function group (eGFR≥60 ml/min/1.73 m^2^)	Impaired kidney function group (eGFR< 60 ml/min/1.73 m^2^)	*P*-value
n (%)	4336 (87.4)	625 (12.6)	
Male	2416 (55.7)	261 (41.8)	< 0.001
Female	1920 (44.3)	364 (58.2)	< 0.001
Mean age, years (SD)	68.9 (12.1)	81.3 (8.9)	< 0.001
Study period years (SD)	6.9 (1.8)	6.3 (2.2)	< 0.001
Mean eGFR screening frequency, days (SD)	300.1 (118.7)	252.9 (111.7)	< 0.001
LDL-cholesterol, mmol/l	2.5 (1.0)	2.4 (1.0)	0.012
HbA1c, mmol/mol	48.7 (13.0)	52.4 (14.1)	< 0.001
eGFR, ml/min/1.73 m^2^	81.1 (18.8)	45.3 (17.9)	< 0.001
Plasma creatinine, μmol/l	77.7 (29.7)	139.5 (109.8)	< 0.001
Hemoglobin, g/l	138.8 (17.9)	127.1 (19.6)	< 0.001
BMI, kg/m^2^	29.9 (5.8)	28.7 (5.1)	0.004
Systolic BP, mmHg	136.0 (18.1)	135.2 (19.9)	0.469
Diastolic BP, mmHg	78.4 (11.2)	75.4 (11.4)	< 0.001
Substantial reduction in eGFR (**≥**25%)	436 (10.8)	146 (25.0)	< 0.001
Any hypertensive medication	3641 (84.0)	585 (93.6)	< 0.001
ACEi/ARB	1714 (39.5)	325 (52.0)	< 0.001
Ca blockers	1115 (25.7)	233 (37.3)	< 0.001
Beta blockers	1890 (43.6)	389 (62.2)	< 0.001
Diuretics	1287 (29.7)	395 (63.2)	< 0.001
Any diabetes medication	3566 (82.2)	490 (78.4)	0.023
Metformin	2975 (68.6)	278 (44.5)	< 0.001
SGLT2i	681 (15.7)	18 (2.9)	< 0.001
GLP-1 RAs	137 (3.2)	15 (2.4)	0.365
Gliptin	1035 (23.9)	279 (44.6)	< 0.001
Glitazone	15 (0.3)	3 (0.5)	0.869
Sulphonylureas	110 (2.5)	20 (3.2)	0.403
Any long-acting insulin	947 (21.8)	224 (35.8)	< 0.001
Multiple daily injections of insulin therapy	537 (12.4)	170 (27.2)	< 0.001
Statin	3060 (70.6)	405 (64.8)	0.004
Ezetimibe	330 (7.6)	38 (6.1)	0.199
Fibrates	21 (0.5)	2 (0.3)	0.802

Note: The *p*-values present the differences between the groups tested with t-test or χ^2^-test. *ACEi*, Angiotensin-Converting Enzyme; *ARB*, Angiotensin Receptor Blockers; *BMI*, Body Mass Index; *DPP-4*, Dipeptidyl-Peptidase-4; *eGFR*, estimated Glomerular Filtration Rate; *GLP-1 RAs*, Glucagon-Like Peptide-1 Receptor Agonists; *HbA1c*, Glycosylated Hemoglobin; *HDL*, High-Density Lipoprotein; *LDL*, Low-Density Lipoprotein; *BP*, Blood Pressure; *SGLT2i*, Sodium-Glucose co-Transporter-2 Inhibitors

The associations between screening frequency and substantial reduction of eGFR in the groups of patients with normal and impaired kidney function are presented in Table 3 and Table 4, respectively. In the normal kidney function group, non-yearly eGFR screening was significantly associated with substantial eGFR reduction in both unadjusted (OR 3.29, 95% CI 2.54–4.33) and adjusted models (OR 2.06, 95% CI 1.21–3.73) compared with yearly screening frequency. In addition, age and prescription of ACEi/ARB were associated with substantial eGFR reduction in both models. However, the association of the achievement of the treatment targets for HbA1c and LDL at baseline and follow-up and the prescription of any long-acting insulin or SGLT2i with this outcome attenuated and became non-significant in the adjusted model.

**Table 3:** Associations of the screening frequency of eGFR and potential covariates with significant eGFR reduction among patients with normal kidney function (eGFR≥60 ml/min/1.73 m^2^)

Normal kidney function group (eGFR ≥ 60 ml/min/1.73 m^**2**^)					
		eGFR reduction			
		< 25%	≥ 25%	OR (unadjusted)	OR (adjusted)
eGFR screened yearly	Yes	1380 (95.2)	69 (4.8)		
	No	2228 (85.9)	367 (14.1)	**3.29 (2.54–4.33,** ***p*** **< 0.001)**	**2.06 (1.21–3.73,** ***p*** **= 0.011)**
Sex n (%)	Male	2013 (89.4)	238 (10.6)	–	–
	Female	1595 (89.0)	198 (11.0)	1.05 (08.6–1.28, *p* = 0.632)	0.98 (0.68–1.40, *p* = 0.900)
Age, mean (SD)		68.8 (11.7)	76.4 (10.4)	**1.07 (1.06–1.08,** ***p*** **< 0.001)**	**1.07 (1.05–1.** 10**,** ***p*** **< 0.001)**
LDL, n (%)	< 2.5 mmol/l	1897 (88.0)	259 (12.0)		
	≥ 2.5 mmol/l	1521 (92.1)	131 (7.9)	**0.63 (0.50–0.79,** ***p*** **< 0.001)**	0.95 (0.65–1.40, *p* = 0.808)
HbA1c, n (%)	< 53 mmol/mol	2605 (91.2)	250 (8.8)	**1.86 (1.50–2.29,** ***p*** **< 0.001)**	
	≥ 53 mmol/mol	904 (84.9)	161 (15.1)		1.48 (0.93–2.34, *p* = 0.100)
systolic BP, n (%)	< 135 mmHg	548 (85.2)	95 (14.8)		
	≥ 135 mmHg	518 (86.5)	81 (13.5)	0.90 (0.65–1.24, *p* = 0.527)	0.72 (0.51–1.02, *p* = 0.069)
ACEi/ARB in use	Yes	2049 (86.9)	310 (13.1)		
	No	1559 (92.5)	126 (7.5)	**1.87 (1.51–2.33,** ***p*** **< 0.001)**	**1.74 (1.16–2.66,** ***p*** **= 0.009)**
SGLT2i in use	Yes	602 (92.8)	47 (7.2)		
	No	3006 (88.5)	389 (11.5)	**0.60 (0.44–0.82,** ***p*** **= 0.002)**	0.70 (0.38–1.24, *p* = 0.244)
GLP-1 RAs in use	Yes	119 (91.5)	11 (8.5)		
	No	3489 (89.1)	425 (10.9)	0.76 (0.38–1.36, *p* = 0.387)	0.92 (0.14–3.66, *p* = 0.912)
Any long-acting insulin in use	Yes	756 (82.8)	157 (17.2)		
	No	2852 (91.1)	279 (8.9)	**2.12 (1.72–2.62,** ***p*** **< 0.001)**	1.40 (0.87–2.23, *p* = 0.162)
Statin in use	Yes	2619 (89.1)	322 (10.9)		
	No	989 (89.7)	114 (10.3)	1.07 (0.85–1.34, *p* = 0.576)	1.02 (0.67–1.58, *p* = 0.937)

*ACEi*, Angiotensin-Converting Enzyme; *ARB*, Angiotensin Receptor Blockers; *eGFR*, estimated Glomerular Filtration Rate; *GLP-1 RAs*, Glucagon-Like Peptide-1 Receptor Agonists; *HbA1c*, Glycosylated Hemoglobin; *LDL*, Low-Density Lipoprotein; *BP*, Blood Pressure; *SGLT2i*, Sodium-Glucose co-Transporter-2 Inhibitors

**Table 4:** Associations of the screening frequency of eGFR and potential covariates with significant eGFR reduction among patients with impaired kidney function (eGFR< 60 ml/min/1.73 m^2^)

Impaired kidney function group (eGFR < 60 ml/min/1.73 m^**2**^)					
		eGFR reduction			
		< 25%	≥ 25%	OR (unadjusted)	OR (adjusted)
eGFR screened yearly	Yes	77 (86.5)	12 (13.5)		
	No	361 (72.9)	134 (27.1)	**2.38 (1.30–4.73,** ***p*** **= 0.008)**	1.89 (0.61–7.21, *p* = 0.301)
Sex n (%)	Male	170 (71.1)	69 (28.9)		
	Female	268 (77.7)	77 (22.3)	0.71 (0.49–1.03, *p* = 0.073)	**0.41 (0.20–0.84,** ***p*** **= 0.016)**
Age, mean (SD)		81.5 (8.9)	82.5 (8.0)	1.01 (0.99–1.04, *p* = 0.201)	**1.06 (1.01–1.12,** ***p*** **= 0.034)**
LDL, n (%)	< 2.5 mmol/l	216 (73.2)	79 (26.8)		
	≥ 2.5 mmol/l	146 (79.3)	38 (20.7)	0.71 (0.45–1. 10, *p* = 0.130)	0.55 (0.24–1.18, *p* = 0.138)
HbA1c, n (%)	< 53 mmol/mol	264 (81.7)	59 (18.3)		
	≥ 53 mmol/mol	135 (64.6)	74 (35.4)	**2.45 (1.65–3.67,** ***p*** **< 0.001)**	0.82 (0.31–2.09, *p* = 0.679)
systolic BP, n (%)	< 135 mmHg	85 (75.2)	28 (24.8)		
	≥ 135 mmHg	97 (76.4)	30 (23.6)	0.94 (0.52–1.70, *p* = 0.835)	0.72 (0.36–1.41, *p* = 0.334)
ACEi/ARB in use	Yes	289 (73.2)	106 (26.8)		
	No	149 (78.8)	40 (21.2)	1.37 (0.91–2.08, *p* = 0.140)	**5.16 (1.94–16.72,** ***p*** **= 0.002)**
GLP-1 RAs in use	Yes	12 (85.7)	2 (14.3)		
	No	426 (74.7)	144 (25.3)	0.49 (0.08–1.84, *p* = 0.358)	0.95 (0.04–8.34, *p* = 0.965)
Any long-acting insulin in use	Yes	131 (61.5)	82 (38.5)		
	No	307 (82.7)	64 (17.3)	**3.00 (2.05–4.43,** ***p*** **< 0.001)**	2.01 (0.81–5.09, *p* = 0.133)
Statin in use	Yes	295 (75.4)	96 (24.6)		
	No	143 (74.1)	50 (25.9)	0.93 (0.63–1.39, *p* = 0.722)	0.59 (0.26–1.36, *p* = 0.210)

*ACEi*, Angiotensin-Converting Enzyme; *ARB*, Angiotensin Receptor Blockers; *eGFR*, estimated Glomerular Filtration Rate; *GLP-1 RAs*, Glucagon-Like Peptide-1 Receptor Agonists; *HbA1c*, Glycosylated Hemoglobin; *LDL*, Low-Density Lipoprotein; *BP*, Blood Pressure

In the impaired kidney function group, only unadjusted non-yearly eGFR screening was significantly associated with substantial eGFR reduction (OR 2.38, 95% CI 1.30–4.73), whereas the adjusted association did not reach statistical significance in the impaired kidney function group (OR 1.89, 95% CI 0.61–7.21). Many of the other variables showed the same attenuating trend, with the achievement of the treatment targets for LDL and HbA1c, alongside SGLT2i and any long-acting insulin, being significantly associated in the unadjusted model but not in the adjusted model. Female sex and ACEi/ARB prescription were significantly associated in the adjusted model (OR 0.41 CI 0.20–0.84 and OR 5.16 CI 1.94–16.72).

## Discussion

### Main findings

In this longitudinal study, we found that annual eGFR screening frequency was associated with significant renal function decline among 5104 primary care T2D patients with normal kidney function. This association was independent of the sex, age, bioclinical variables, and prescribed antihyperglycemic and cardiovascular medications. A similar trend in the association between annual eGFR screening and significant renal function decline was observed in the impaired kidney function group.

### Screening frequency and current guidelines

To the best of our knowledge, earlier studies have not investigated the association of eGFR screening frequency with renal function decline among patients with T2D. Although international guidelines recommend the annual screening of CKD in patients with T2D and more frequent monitoring of kidney function if CKD is diagnosed, these guidelines are derived from expert opinion and indirectly from renoprotective medication research [2, 13, 14]. This study improves the existing knowledge on the role of annual eGFR screening in significant renal function decline in a real-life setting. Our finding supports the current guidelines regarding annual eGFR screening in T2D patients, which earlier studies have shown to be lacking in 7–22% of patients [9, 13, 15, 19, 23].

Yearly eGFR screening was associated with renal function decline in patients with normal kidney function. In the present study, we used a cut-off level of 25% as the substantial reduction in eGFR, in accordance with KDIGO guidelines [2]. This further underscores the meaning of our novel findings. In addition to well-established confounders of the present study, the observed results could be attributed to early medical treatment with proper patient education and better overall compliance to treatment [13]. A similar association was noted in patients with impaired kidney function. However, after adjusting for confounders, the association did not reach statistical significance. One might speculate that the attenuation in the impaired kidney function group was due to the small patient sample size. Additionally, the heterogeneity (eGFR ranging from mildly impaired kidney function to ESKD) in the corresponding group may result in variability concerning treatment, medication usage, and monitoring. Moreover, patients with eGFR< 30 ml/min/1.73 m^2^ are more likely to be monitored more closely and treated by a nephrologist than a primary care doctor [2, 13].

### Strengths and weaknesses

The main strengths of our study are the real-life primary care setting that captures the majority of the T2D patients in the region, the large population size, and the longitudinal design. In Finnish health care, electronic patient registry data and electronic medicine prescriptions are extensively used as sources of comprehensive medical data. All of the patient samples were taken, processed, and analyzed by the same laboratory, avoiding possible discrepancies in the results. Concurrently, the study has several limitations that need to be considered while interpreting our results. Data on the patients treated and monitored by nephrologists (e.g., patients with severely decreased kidney function or ESKD) were not available. The majority of CKD patients with T2D are diagnosed and treated in primary care by general practitioners in European countries, with only patients with severely decreased eGFR and moderately to severely increased albuminuria requiring referral to a nephrologist. The findings of the current study are derived from a single center, and mainly of older patients and therefore should not be directly generalized to other patient groups*.* The changes in medication therapy on control visits, smoking status, physical activity, socio-economic status, and patient mortality were also not available for analysis, which can be considered a limitation of our study.

## Conclusion

In conclusion, the novel findings of the present study highlight the role of regular eGFR screening in the prevention of kidney function decline. This confirms the importance of regular eGFR screening in clinicians’ daily practice. Further studies are needed to determine the association of the screening frequency of albuminuria with renal function decline in the prevention of renal impairment in T2D patients, especially in primary health care settings.

## Data Availability

The data that support the findings of this study are available from Rovaniemi City Health Services, but restrictions apply to the availability of these data, which were used under license for the current study, and so are not publicly available. Data are however available from the corresponding author, Maria Hagnäs upon reasonable request and with permission of Rovaniemi City Health Services.
